# Supplementary material for: α‑1,3-Glucan-Driven Remodeling of the Conidial Cell Wall in an Aspergillus fumigatus Vaccine Strain Alters Innate Immune Recognition
Source: J Am Chem Soc. 2026 Mar 26;148(13):14320–32. doi: 10.1021/jacs.6c00915 (PMC13067271; doi:10.1021/jacs.6c00915)
Supplement: Supplementary file 1 [file ja6c00915_si_001.pdf]

Supplementary Information

α-1,3-Glucan-Driven Remodeling of the Conidial Cell Wall in an *Aspergillus fumigatus* Vaccine Strain Alters Innate Immune Recognition

Kalpana Singh[1#], Ankur Ankur[1#], Jayasubba Reddy Yarava[1], Caroline Mota Fernandes[2], Gianluca Vascelli[3], Alessia Sulla[3], Teresa Zelante[3], Maurizio Del Poeta[2,4,5*], and Tuo Wang[1,*]

[1] Department of Chemistry, Michigan State University, East Lansing, MI 48824, USA

[2] Department of Microbiology and Immunology, Stony Brook University, Stony Brook, NY 11794, USA

[3] Department of Medicine and Surgery, University of Perugia, Perugia, 06123, Italy

[4] Division of Infectious Diseases, Stony Brook University, Stony Brook, NY 11794, USA

[5] Veterans Affairs Medical Center, Northport, NY 11768, USA

[#]These authors contributed equally

[*]Correspondence: maurizio.delpoeta@stonybrook.edu; wangtuo1@msu.edu

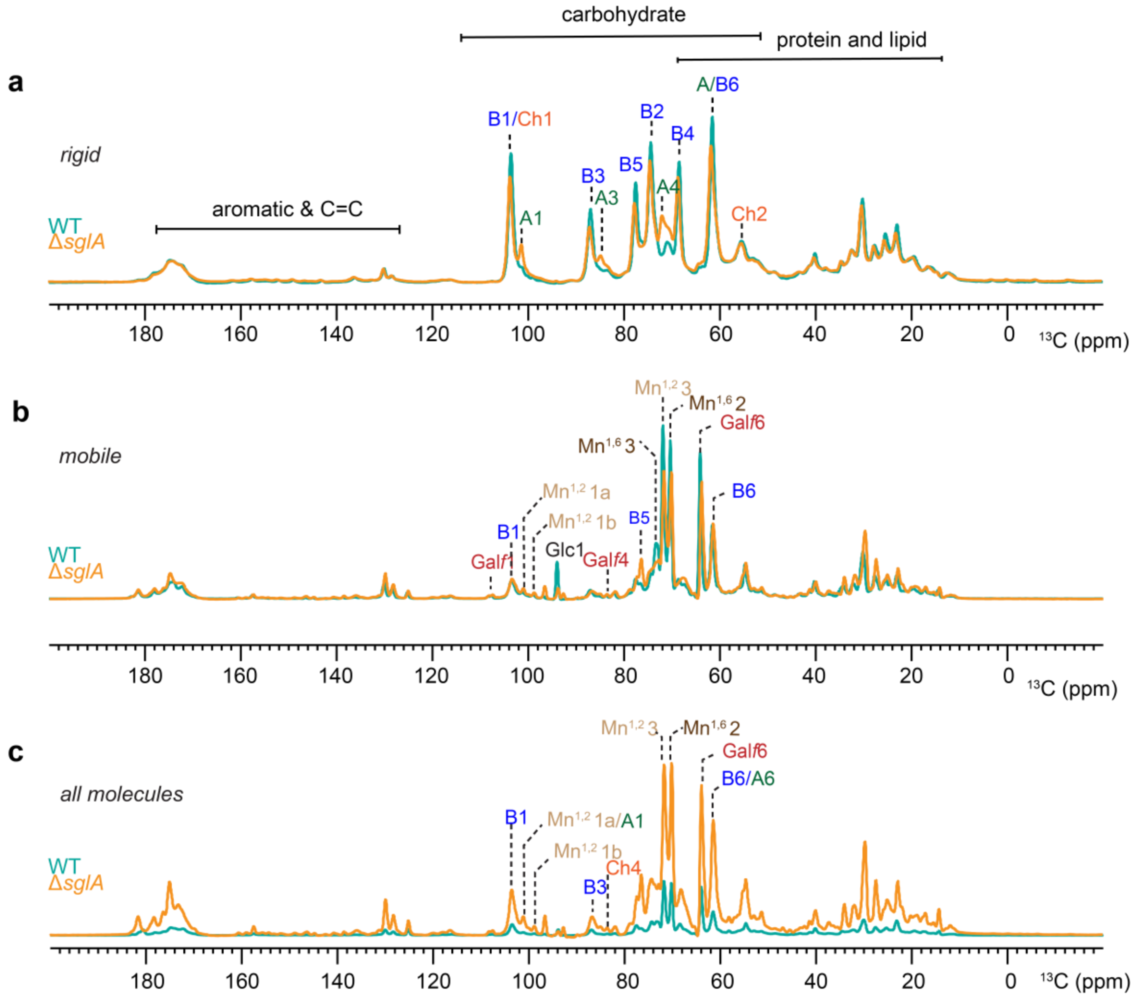

Figure S1. 1D ^{13}C spectra of intact *A. fumigatus* conidia. The spectra are color coded to show the cyan spectra for WT and yellow for mutant Δ*sglA*. (**a**) Carbohydrates showing the rigid glucans obtained by CP experiment. (**b**) The mobile glucans probed by 2 s DP spectra and (**c**) The quantitative glucans represented by the 35 s DP spectra.

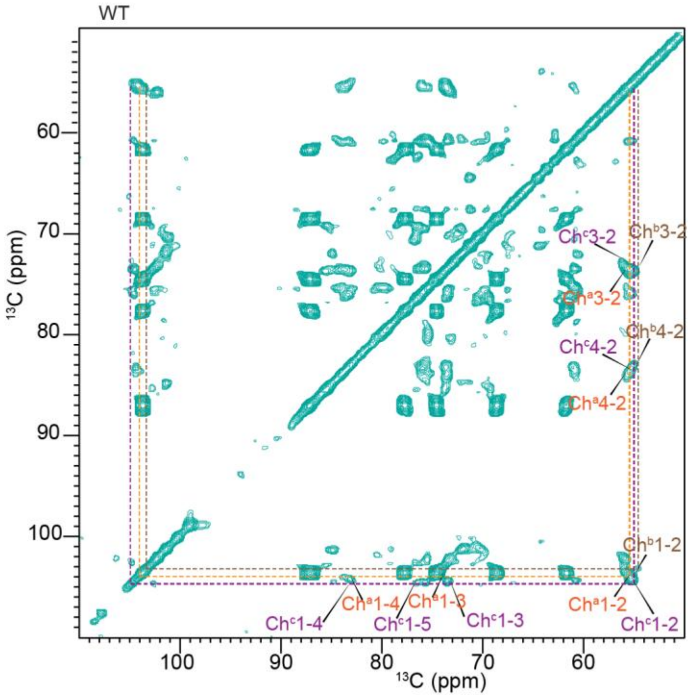

Figure S2. 2D ^{13}C-^{13}C CORD spectra showing the rigid components in *A. fumigatus* WT conidia. It shows the presence of polymorphic forms of chitin.

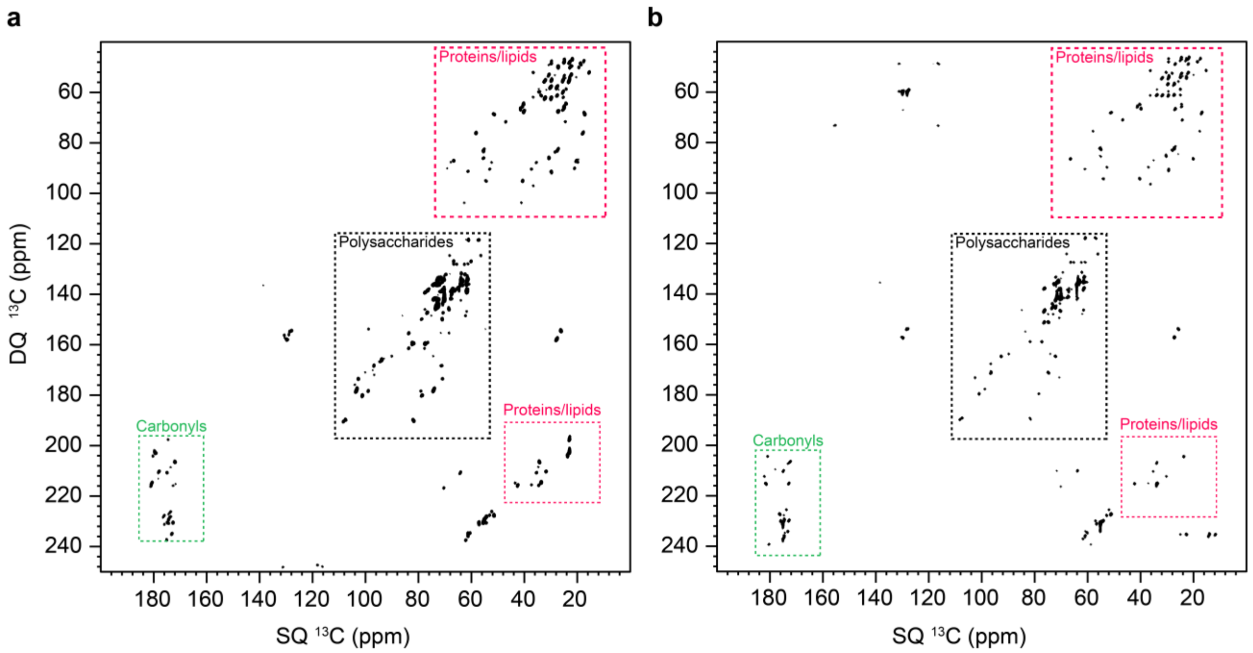

Figure S3. Biopolymer signals in 2D ^{13}C refocused DP J-INADEQUATE. Mobile polysaccharide components of the cell wall in *Aspergillus* (**A**) wild-type Δ*akuB*^{KU80} and (**B**) Δ*sglA* mutant probed using 2D DP ^{13}C refocused J-INADEQUATE spectrum. The individual regions were marked with quadrangles of different colors: green for carbonyls, black for polysaccharides, and red for aliphatics from proteins and lipids. The individual regions are marked using the same color code mentioned above.

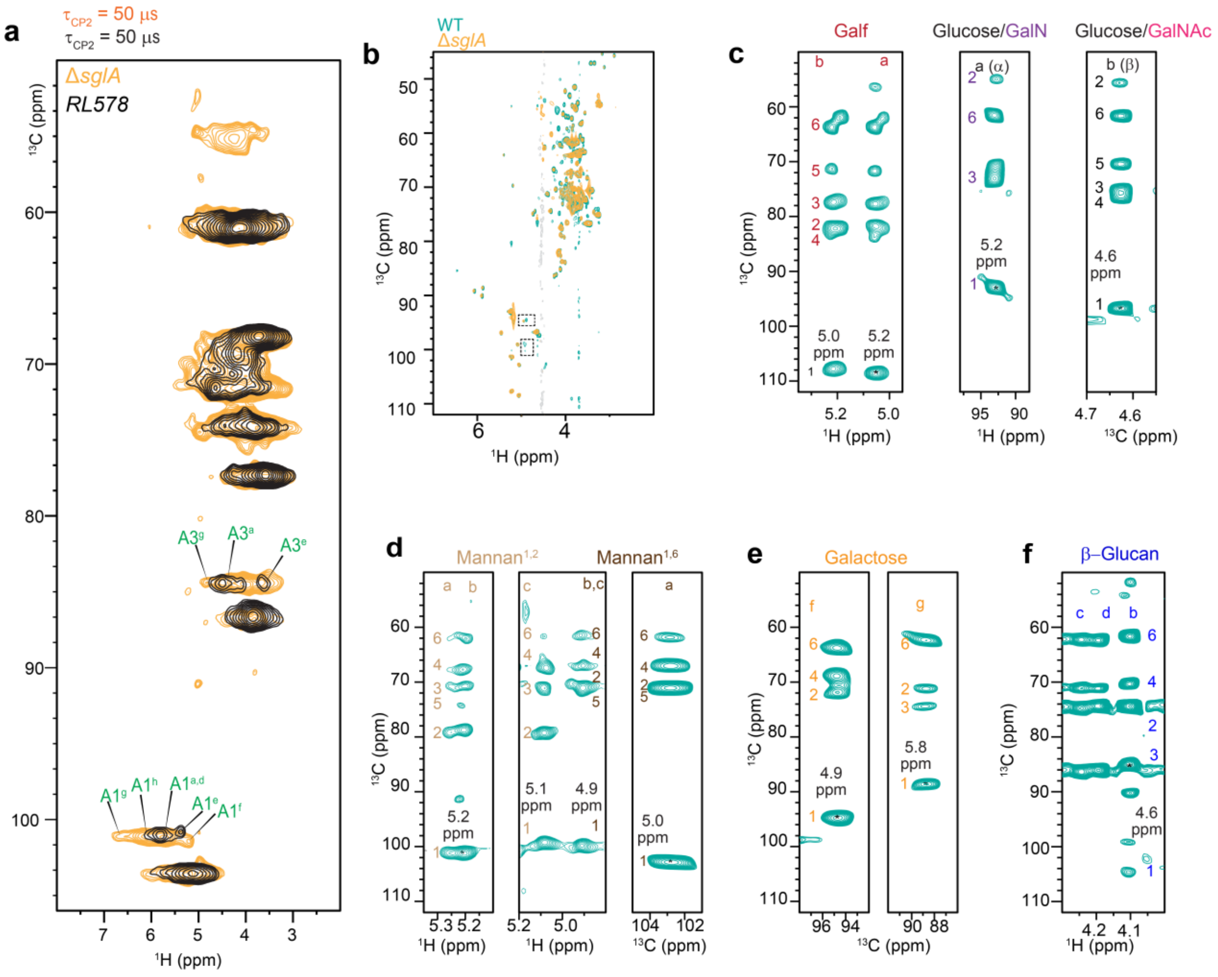

Figure S4. Identifying polymorphic forms of rigid polysaccharides in of *A. fumigatus* conidial wall. (**a**) Comparison of the resolved polymorphic forms of α-1,3-glucan in the fully protonated conidial cell wall of mutant Δ*sglA* and the partially deuterated mycelial cell wall of the *RL578* wild-type cells. The polymorphic forms a (^{13}C:101.1 and ^{1}H:5.7 ppm), d (^{13}C: 101.1 ppm, ^{1}H: 5.7 ppm), e (^{13}C: 100.76 ppm, ^{1}H: 5.4 ppm) and h (^{13}C: 101.1 ppm, ^{1}H: 6.0 ppm) were matched. Additionally, two novel forms, designated as f (^{13}C: 101.42 ppm, ^{1}H: 5.2 ppm) and g (^{13}C: 101.0 ppm, ^{1}H: 6.58 ppm) were detected in Δ*sglA* mutant. (**b**) Overlaid carbohydrate regions of the 2D hcCH TOCSY (DIPSI-3) spectra of *A. fumigatus* wild-type (WT) (green) and the Δ*sglA* (mutant) (orange) cell walls, showing one-bond ^{13}C-^{1}H correlations. Water singles are shown in grey. Dotted squares highlight signals present in the WT but absent in the Δ*sglA* mutant, indicating specific carbohydrate components affected by the gene deletion. The strips were extracted from the 3D hCCH TOCSY (DIPSI-3) spectrum and show through-bond carbon-carbon connectivity. The strip extracted at a specific carbon or proton site is indicated by an asterisk (*). Resolved signals assigned to: (**c**) two forms of glucofuranose (Gal*f*), one form of galactosamine (GalN), and one form of N-acetyl galactosamine (GalNAc); (**d**) three forms each of α-1,2-mannan and α-1,6-mannan (**e**) seven forms of galactose derivatives; (**f**) four forms of β-glucan; Carbon positions are numbered (1-6), and lowercase letters denote different allomorphic forms. All spectra of were acquired on a 14.1 T spectrometer at a MAS rate of 60 kHz and (c) were on 800MHz at MAS of 15 kHz.

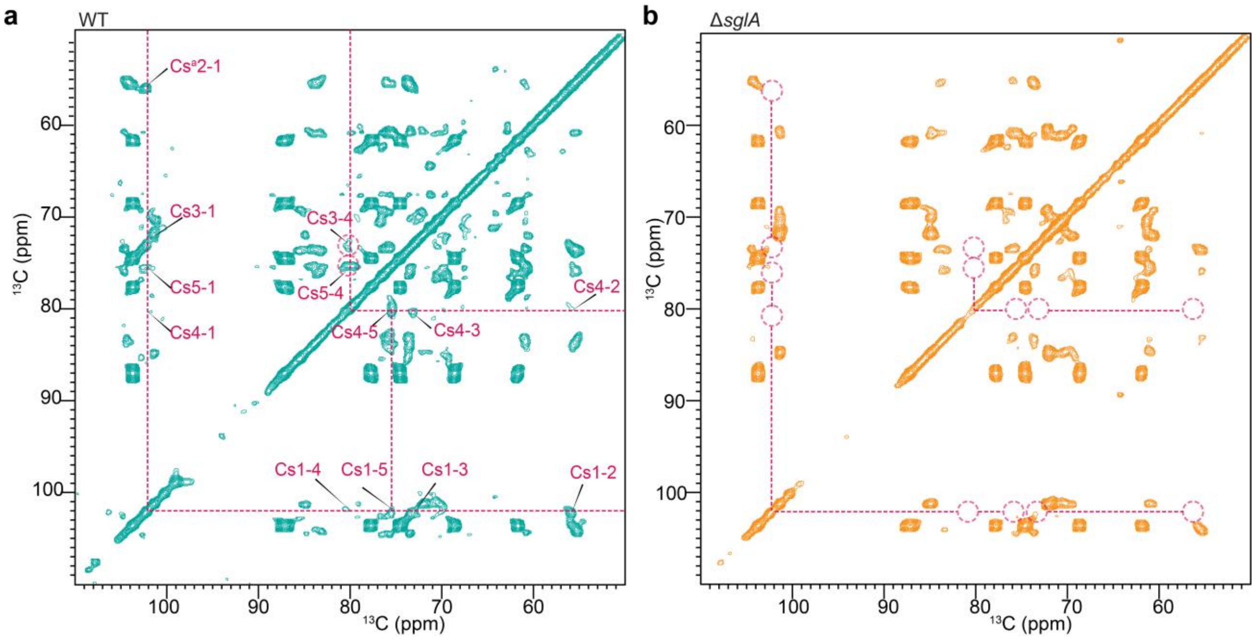

Figure S5. (**a**) 2D ^{13}C-^{13}C CORD spectra showing the rigid components in *A. fumigatus* WT conidia (cyan). It shows the presence of chitosan. (**b**) 2D ^{13}C-^{13}C CORD spectra showing the rigid components for mutant Δ*sglA* in orange spectra. It shows the absence of chitosan. All measurements were obtained using 800 MHz NMR spectrometer at 15 kHz.

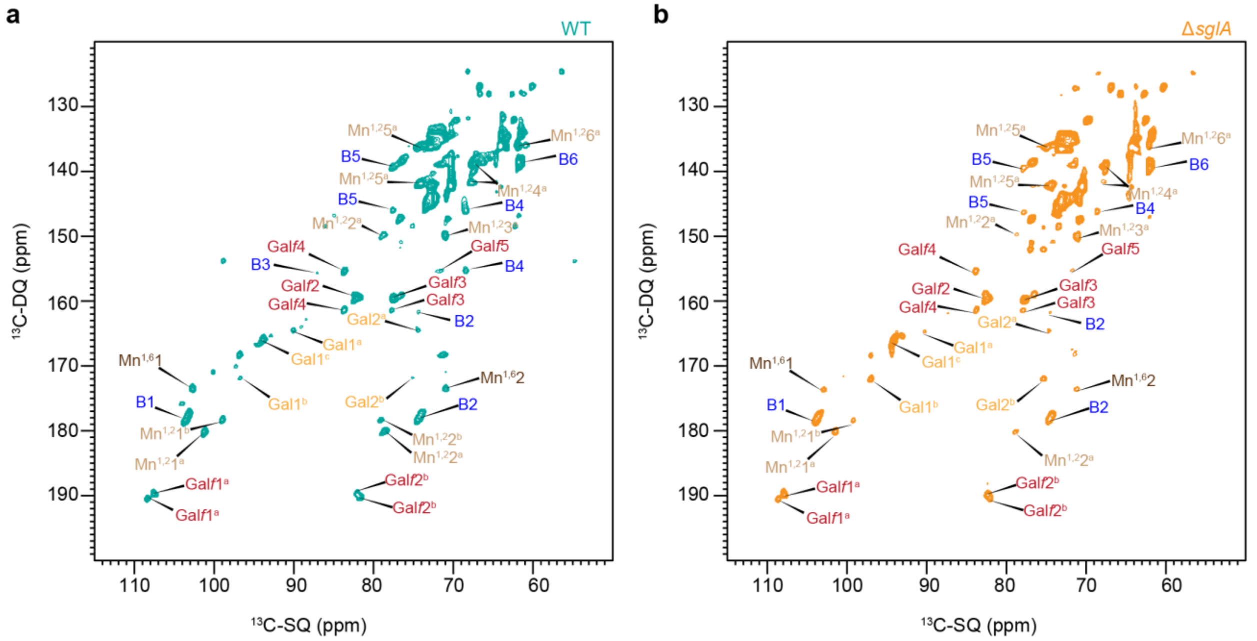

Figure S6. Mobile carbohydrates in *Aspergillus* conidia. (**a**) The 2D ^{13}C-DP INADEQUATE spectra of WT conidia showing all the mobile glucans. (**b**) The mobile spectra representing mutant Δ*sglA* showing decrease in the intensity of mannose 1,2. The spectra of WT and mutant Δ*sglA* were measured on 800MHz at 15 kHz.

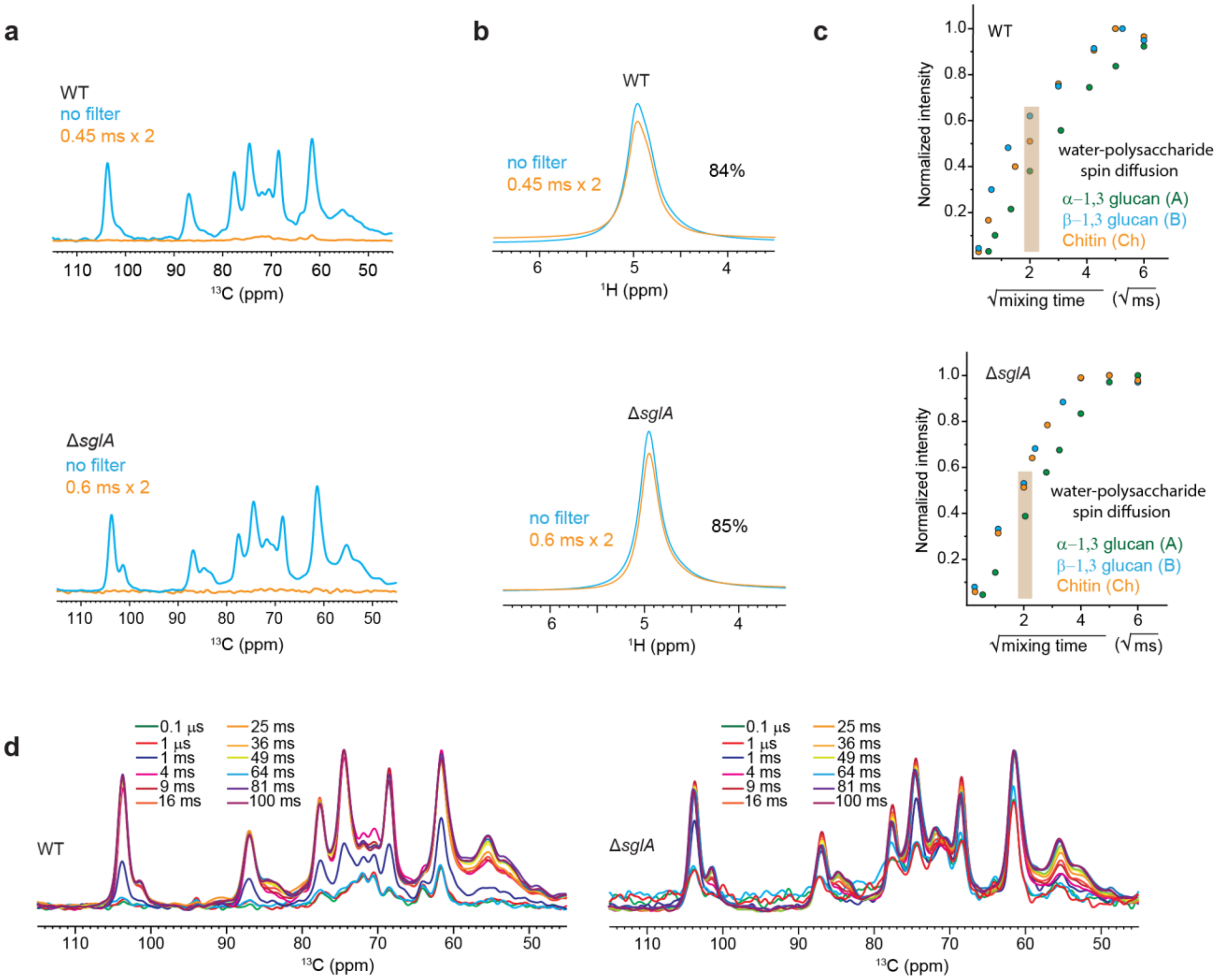

Figure S7. Water-edited experiments to monitor carbohydrate-associated water. (a) ^{1}H-T$_2$ filtered (orange) and control(blue) ^{13}C spectra are shown for WT (top) and mutant (bottom). **(b)** ^{1}H-T$_2$ filtered (orange) and control (blue) ^{1}H NMR spectra, with 84% and 85% of water signal retained for both samples after the ^{1}H-T$_2$ filter**. (c)** Representative water-to-polysaccharide ^{1}H spin diffusion buildup curves. **(d)** 1D water-edited spectra with different ^{1}H mixing times are shown for WT and mutant samples. All the spectra were measured on 400MHz spectrometer at 10 kHz at 277K.

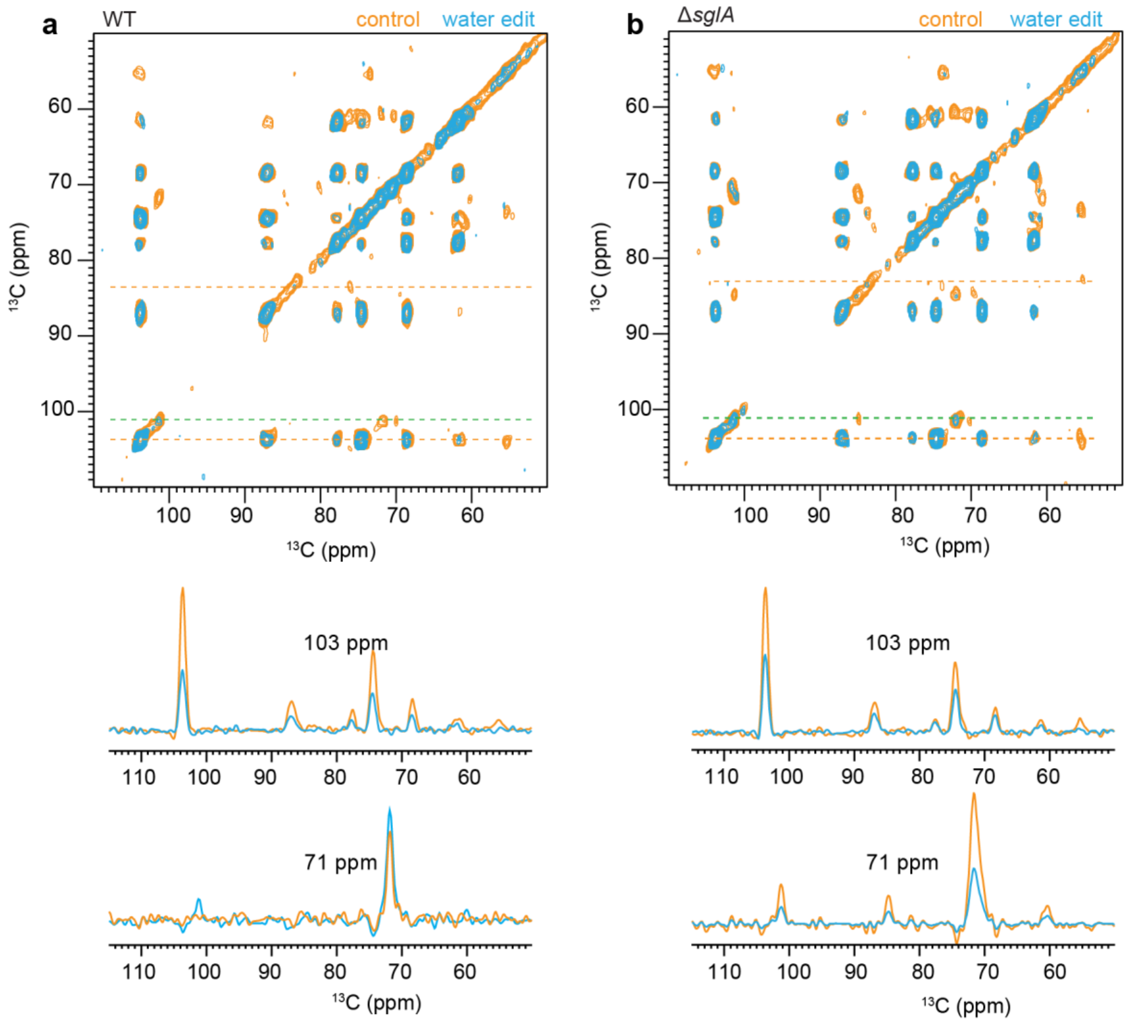

Figure S8. Water-edited analysis for *Aspergillus* fumigatus conidial cell wall. (**a**) Overlay of 2D water-edited (blue) and control (orange) ^{13}C-^{13}C correlation spectra (T2 = 0.45 ms × 2 for Sample. Representative 1D slices extracted from the 2D ^{13}C-^{13}C correlation spectra are shown for WT (left bottom) sample (**b**) Overlay of 2D water-edited (blue) and control (orange) ^{13}C-^{13}C correlation spectra (T_2 = 0.6 ms × 2 for Sample. Representative 1D slices extracted from the 2D ^{13}C-^{13}C correlation spectra are shown for mutant (right bottom) sample All the measurements were taken on a 400 MHz spectrometer at 10 kHz MAS and 277 K.

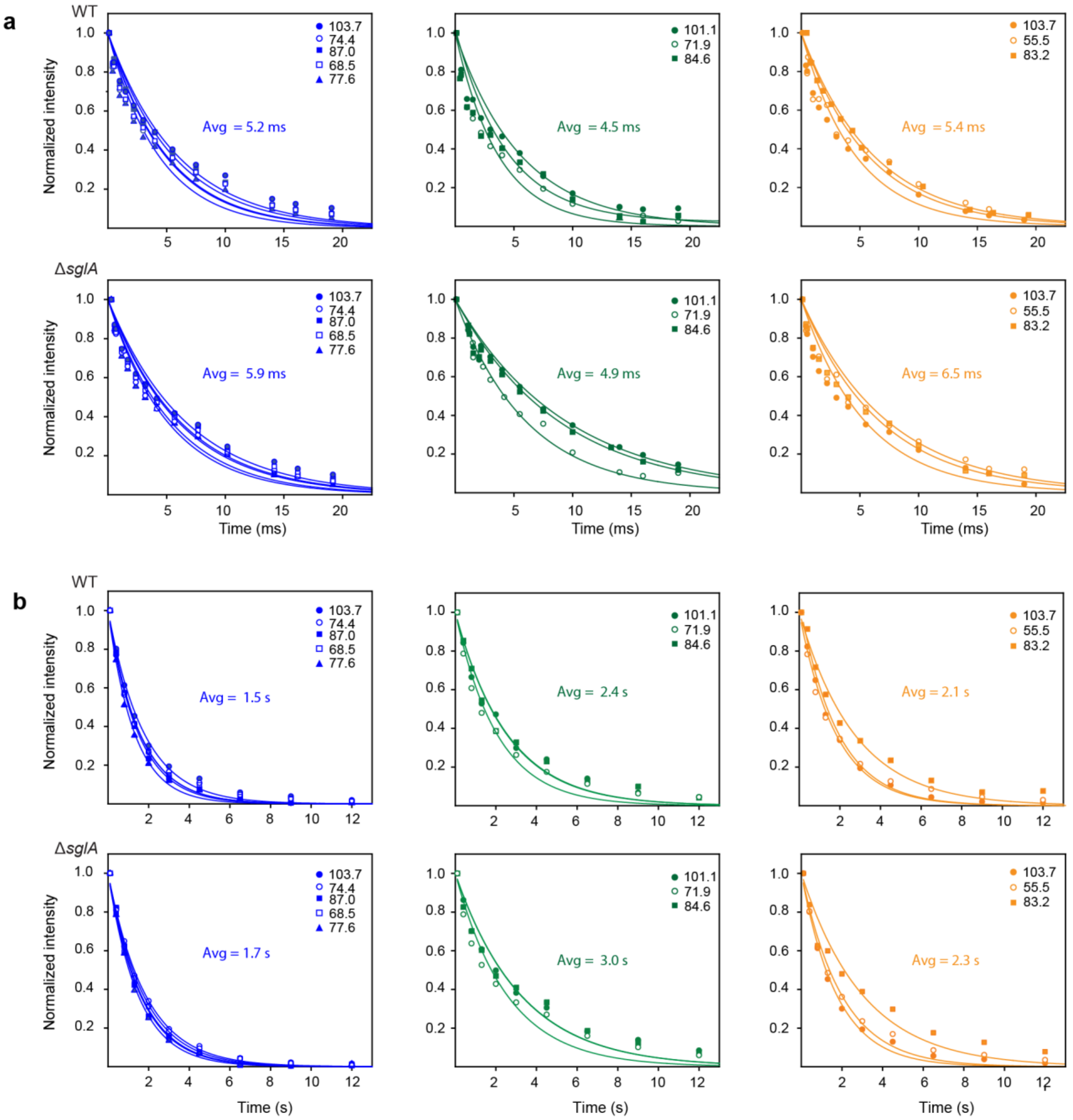

Figure S9. Relaxation curves of polysaccharides in *A. fumigatus* conidial cell walls. The relaxation decay curves are shown separately for (**a**) ^{1}H-$T_{1\rho}$ and (**b**)^{13}C-T_1 of the *A. fumigatus* WT and mutant Δ*sglA* conidia.

Table S1. Average cell thickness of *A. fumigatus* conidial cells. The results are the average and standard deviation of measurements from SEM images of different cells (n=9 for WT and Δ*sglA*, n=9).

Strain	Average cell thickness (μm)
WT	1.8 ± 0.2
Δ*sglA*	1.9 ± 0.2

Table S2. Molar composition of rigid polysaccharides in *A. fumigatus* conidial cell wall. The numbers were calculated using the integrals of well-resolved cross peaks of β-1,3 glucan, α-1,3 glucan, β-1,6 glucan, chitosan and chitin in 2D ^{13}C-^{13}C CORD spectra. The results were already normalized by the number of scans.

Sample	α-1,3-glucan	β-1,3-glucan	Chitin	β-1,6-glucan	Chitosan
WT	2 ± 1%	78 ± 12%	11 ± 3%	4 ± 0.9%	5 ± 1%
Δ*sglA*	20 ± 4%	67 ± 10%	8 ± 2%	5 ± 2%	NA

The area of the following well-resolved cross peaks 53 ms CORD spectra are used:
β-1,3 (a): the average of C1-C2/3/4/5 and C3-C2/4/5/6.
β-1,6: the average of C3/5-C4 and C5-C6.
Chitin: the average of C1-2/4/5, C3-C2, C4-C2/3/5, and C5-C2.
Mannan: the average of C2-C5 and C2-C6.

Table S3. **Molar composition of mobile polysaccharides in *A. fumigatus* conidial cell wall.** The numbers were calculated using the integrals of well-resolved cross peaks of β-1,3 glucan, mannose units, and glucofuranose units, in ^{13}C DP *J*-INADEQUATE spectra. The results were already normalized by the number of scans.

Sample	β-1,3-glucan	$Mn^{1,2}$	$Mn^{1,6}$	Gal*f*
WT	22 ± 5%	26 ± 4%	22 ± 3%	30 ± 8%
sglA	46 ± 6%	17 ± 2%	13 ± 3%	24 ± 8%

The area of the following well-resolved cross peaks of 2D ^{13}C *J*- INADEQUATE spectra are used:
β-1,3-glucan: the average of C1-C2, C2-C3 and C5-C6
Gal*f*: the average of C1-C2, C2-C3, C3-C4 and C4-C5
Mannan: the average of C1-C2, C2-C3 and C3-C4

Table S4. Water-edited intensities of the polysaccharides. The intensity ratios are obtained by comparing the peak intensities between water-edited and the control 2D spectra of the *Aspergillus* conidial cell walls. The error bars represent propagated standard deviations from NMR signal-to-noise ratios.

β-1,3-glucan				α-1,3-glucan				Chitin			
(ω1 ppm, ω2 ppm)		WT (S/S_0)	Δ*sglA* (S/S_0)	(ω1 ppm, ω2 ppm)		WT (S/S_0)	Δ*sglA* (S/S_0)	(ω1 ppm, ω2 ppm)		WT (S/S_0)	Δ*sglA* (S/S_0)
103 ppm	103.6	0.71±0.03	0.43±0.02	101 ppm	101.6	0.35±0.08	0.38±0.08	103 ppm	103.6	0.71±0.07	0.43±0.02
	86.7	0.82±0.15	0.51±0.13		84.6	0.23±0.09	0.34±0.05		55.3	0.31±0.01	0.32±0.05
	77.4	-	0.54±0.19		71.6	0.44±0.07	0.35±0.07		73.1	0.29±0.01	-
	74.3	0.81±0.06	0.48±0.04		69.6	0.64±0.07	-		76.3	0.5±0.0	-
	68.1	0.89±0.19	0.51±0.12		60.2	0.85±0.11	-		82.5	0.36±0.02	0.61±0.14
	61.1	0.75±0.35	0.68±0.09								
86 ppm	103.5	0.81±0.12	0.62±0.10	84 ppm	101.1	0.41±0.03	0.59±0.12	83 ppm	103.6	0.13±0.03	-
	86.6	0.79±0.08	0.47±0.06		84.5	0.24±0.10	0.40±0.02		55.3	0.35±0.02	0.60±0.01
	77.3	0.86±0.13	0.67±0.12		71.6	0.57±0.03	-		73.6	0.50±0.05	-
	74.3	0.84±0.11	0.52±0.08		69.3	0.61±0.05	0.31±0.08		75.9	0.56±0.05	0.45±0.09
	68.2	0.78±0.12	0.51±0.09		60.2	0.37±0.6	0.24±0.07		83.4	0.19±0.03	0.41±0.06
	61.1	-	0.86±0.10								
77 ppm	103.5	0.94±0.4	0.59±0.18	71.4 ppm	101.2	0.42±0.18	0.16±0.05	55 ppm	103. 3	0.59±0.03	0.55±0.14
	86.71	0.74±0.26	0.34±0.16		84.7	0.19±0.02	0.34±0.02		54.7	0.31±0.01	0.32±0.06
	77.1	0.98±0.08	0.52±0.04		71.6	0.48±0.05	0.65±0.006		73.6	0.42±0.01	-
	74.4	0.88±0.47	0.56±0.11		60.3	0.58±0.06	0.43±0.08		75.4	0.68±0.09	-
	68.1	0.79±0.10	0.53±0.07		69.3	0.63±0.09	0.35±0.06		83.4	0.74±0.02	0.42±0.11
	61.2	-	0.51±0.06								
74 ppm	103.5	0.84±0.10	0.49±0.04								
	86.6	0.87±0.1	0.55±0.11								
	77.2	-	0.48±0.11								
	74.2	0.68±0.11	0.49±0.11								
	68.2	0.84±0.12	0.52±0.13								
	61.0	0.72±0.10	0.50±0.14								
68 ppm	103.6	0.74±0.15	0.51±0.12								
	86.6	0.82±0.17	0.49±0.11								
	77.4	0.77±0.13	0.53±0.07								
	74.3	0.60±0.11	0.52±0.10								
	68.5	0.74±0.04	0.49±0.03								
	61.1	0.74±0.14	0.43±0.11								
	n	26	30			15	12			15	9
Average		0.81	0.53			0.47	0.35			0.53	0.46

Table S5. ^{13}**C-T$_1$ and ^{1}H-T$_{1\rho}$ relaxation time constants of polysaccharides in *A. fumigatus*.** A single exponential equation was used to fit the T1 data $I(t) = e^{-t/T_I}$. A single exponential equation was used to fit the T$_{1\rho}$ data: $I(t) = e^{-t/T_{I\rho}}$. Error bars are standard deviations of the fit parameters.

Sample type	Cross peaks	T_1 (s)	Average	Cross peaks	$T_{1\rho}$ (ms)	Average
WT	B1	1.8±0.1	1.5	B1	6.1±0.5	5.2
	B2	1.5±0.1		B2	5.6±0.4	
	B3	1.3±0.1		B3	4.4±0.4	
	B4	1.5±0.1		B4	5.0±0.4	
	B5	1.5±0.1		B5	5.1±0.4	
	A1	2.6±0.1	2.4	A1	4.3±0.6	4.5
	A2/A5	2.5±0.1		A2/A5	5.6±0.8	
	A3	2.0±0.1		A3	3.7±0.5	
	Ch1	1.9±0.1	2.1	Ch1	6.1±0.5	5.4
	Ch2	1.8±0.1		Ch2	4.6±0.5	
	Ch4	2.8±0.2		Ch4	5.6±0.7	
Δ*sglA*	B1	1.9±0.1	1.7	B1	6.7±0.7	5.9
	B2	1.6±0.1		B2	6.2±0.6	
	B3	1.5±0.1		B3	5.1±0.5	
	B4	1.8±0.1		B4	5.4±0.5	
	B5	1.6±0.1		B5	6.1±0.6	
	A1	3.2±0.1	3.0	A1	5.8±0.1	4.9
	A2/A5	3.2±0.2		A2/A5	5.4±0.1	
	A3	2.6±0.1		A3	3.4±0.9	
	Ch1	1.8±0.1	2.3	Ch1	6.7±0.7	6.5
	Ch2	2.0±0.1		Ch2	5.4±0.7	
	Ch4	3.0±0.1		Ch4	7.4±0.9	

Table S6. **Recipe of mineral-based solid medium.** The pH is adjusted to 6.5 with H_3PO_4 or 0.25 M KOH. Each sample uses 100 mL of medium that contains 2g agar, 2 g of ^{13}C-glucose and 5 mL of sodium nitrate solution with 0.1ml of trace elements. The culture media and condition were adapted from a previously described protocol[1].

	Reagent	Quantity for 1L
Trace elements	$ZnSO_4.7H_2O$	22.0g
	H_3BO_3	11.0g
	$MnCl_2.4H_2O$	5.0g
	$FeSO_4.7H_2O$	5.0g
	$CuSO_4.5H_2O$	1.6.0g
	$CoCl_2.6H_2O$	1.6.0g
	$(NH_4)_6Mo_7O_{24}.4H_2O$	1.1.0g
	EDTA	50.0g
Nitrate salt solution	$NaNO_3$	300.0g
	KCl	26.0g
	$MgSO_4.7H_2O$	24.0g
	KH_2PO_4	120
	K_2HPO_4	20.9
Carbon source	Glucose	10.0

Table S7. **^{13}C chemical shifts of *A. fumigatus* conidial cell wall.** For each carbon site, the ^{13}C chemical shifts are shown in the top and bottom rows, respectively. The referencing scale is TMS scale for ^{13}C, with ambiguity are underlined:

Carbohydrates	forms	C1	C2	C3	C4	C5	C6	Reference
Rigid molecules								
β-1,3-glucan (B)		103.6	74.4	86.8	68.4	77.6	61.4	Shim *et al*. 2007[2]
α-1,3-glucan	a	101.2	71.9	84.9	69.9	71.6	60.9	Bhanja et al.2014[3]
	f	101.6	70.8	85.1	69.7	71.9	60.6	
Chitosan		102.2	55.9	73.0	80.4	75.7	-	
Chitin	a	103.9	55.3	73.7	83.5	75.6	60.7	Fernando *et al.* 2021[4]
	b	103.2	54.4	73.4	82.9	75.6	60.8	
	c	104.5	55.0	73.5	83.5	75.6		
Mannan		102.3	71	63.9	67.1	75.8	60.6	
β-1,6-glucan	-	-	-	76.7	70.5		69.4	Lowman *et al.* 2011 [5]
Mobile molecules								
β-1,3-glucan (B)		103.6	74.4	86.7	68.3	77.6	61.5	Shim *et al.* 2007[6]<br>Fairweather *et al.* 2009[7]<br>Saito *et al.* 1979[8]
β-1,5 galactofuranose (Gal*f*)	a	108	81.8	77.5	83.5	71.7	63.5	Chakraborty *et al.* 2021[9]
	b	107.5	81.9	77.4				
α-1,6-Mannan ($Mn^{1,6}$)		102.7	70.8	73.8	67.7	70.8	---	Latge *et al.* 1994[10]<br>Chakraborty *et al.* 2021[9]
α-1,2-Mannan ($Mn^{1,2}$)	a	101.1	78.5	70.9	67.8	74.0	62.1	
	b	98.9	79.0	71.2	67.0	73.4	62.0	
Galactose/Glucose or their derivatives (Gl)	a	90.0	<u>74.0</u>	<u>74.5</u>	<u>70.3</u>	---	61.8	Fontaine *et al.* 2011[11]
	b	97.0	<u>71.1</u>	<u>74.4</u>	<u>67.0</u>	---	61.8	Archbald et al. 1981[12]
	c	94.7	<u>72.5</u>	<u>73.6</u>	<u>67.4</u>	---	61.9	Archbald et al. 1981[12]<br>Fontaine *et al.* 2011[11]

Table S8. Solid-state NMR experiments and parameters. To be quantitative, direct pulse (DP) experiments with 35 s long recycling delay were used. cross polarization (CP), most rigid molecules. With DP and a shorter recycling delay of 2 seconds, suppress the rigid molecules from the spectra, and with Insensitive Nuclei Enhanced by Polarization Transfer (INEPT) the most mobile molecules were selected. For 2D ^{13}C-^{13}C correlation experiments allowed to resolve rigid intramolecular peaks. 2D DQ-SQ, DP J-INADEQAUTE spectra was used to detect through-bond correlations. The experimental parameters include the ^{1}H Larmor frequency, total experiment time (t), recycle delay (d1), number of scans (NS), The number of points for the direct (td2) and indirect (td1) dimensions, the acquisition time of the direct dimension (aq2) and the evolution time of indirect dimension (aq1), spectral width (sw1 and sw2).

Experiment	B_0 (T)	t (h)	d1 (s)	NS	td2	td1	aq2 (ms)	aq1 (ms)	sw2 (ppm)	sw1 (ppm)
1D ^{13}C CP	18.8	0.5	1.8	1024	3600		18		496.8	
1D ^{13}C DP	18.8	0.5	2.0	256	3600		18		496.8	
1D ^{13}C DP	18.8	2.5	35.0	256	3600		18		496.8	
1D ^{13}C refocused INEPT	18.8	1.5	4.0	1024	3200		16		496.8	
2D ^{13}C-^{13}C with CORD mixing	18.8	11	2.0	32	2800	600	14	7.5	496.8	198.7
2D ^{13}C refocused DP J-INADEQUATE	18.8	6	1.5	16	2600	1024	19	10	326.8	248.5
1D ^{13}C T_1	9.4	3.5	2.0	512	2000		16		623.3	
1D ^{1}H $T_{1\rho}$	9.4	4.0	2.0	512	2000		16		623.3	
2D ^{13}C-^{13}C water-edited	9.4	8.0	2.0	64	2000	220	16	5.4	623.3	199.4

Table S9. ^{1}H and ^{13}C chemical shifts of *A. fumigatus* mobile polysaccharides from ^{1}H-detection experiments. For each carbon site, the ^{13}C and ^{1}H chemical shifts are shown in the top and bottom rows, respectively. The referencing scale is TMS scale for ^{13}C, and DSS for ^{1}H. ^{13}C and ^{1}H sites with ambiguity are underlined for Gl, the carbon numbering of C2, 3, 4 is uncertain.

	forms	C1	C2	C3	C4	C5	C6	Reference
Rigid molecules								
β -1,3-glucan (B)	a	---	---	85.34<br>3.95	---	---	---	Shim *et al.* 2007[2]
	b	104.5<br>4.6	74.4<br>3.5	84.75<br>4.1	70.34<br>3.6	-	61.7<br>3.7,3.8	Fairweather *et al.* 2009[7]
	c	---	74.5<br>3.5/4.3	84.75<br>4.25	70.86<br>---	---	62.36<br>3.8,3.9	Saito *et al.* 1979[8]
	d	---	74.3	86<br>4.19	71.05	---	62.5<br>3.8,3.9	
α-1,6-Mannan ($Mn^{1,6}$)	a	102.8<br>5.04	71.00<br>4.07/3.45	---	67.0<br>3.6/3.8	71.00<br>4.07/3.45	61.85<br>3.7/3.8	Latge *et al.* 1994[13]
	b	99.10<br>4.9	71.02<br>3.9	72.5<br>3.8/3.5	67.04<br>3.93	71.02<br>3.9	61.44<br>3.7/3.8	Chakraborty *et al.* 2021[14]
	c	100.25<br>4.9	70.2<br>3.9	---	67.04<br>3.93	70.2<br>3.9	61.44<br>3.7/3.8	Kuraoka *et al.* 2021[15]
α-1,2-Mannan ($Mn^{1,2}$)	a	101.3<br>5.2	78.67<br>4.09	70.90<br>3.95	67.7<br>3.65	74.04	61.90<br>3.80/3.7	Kuraoka *et al.* 2021[15]
	b	101.3<br>5.2	78.67<br>4.09	70.9<br>3.95	67.70<br>3.65	74.04	61.9<br>3.88,3.70	Kuraoka *et al.* 2018[16]
	c	99.07<br>5.08	79.34<br>4.01	71.12<br>3.95	67.24<br>3.79	---	61.79<br>3.60/ 3.80	
Galactose/Glucose or their derivatives (GI)	a	90.00<br>5.88	---	74.4<br>4.30	70.30<br>4.20	---	61.70<br>3.88/ 3.78	Fontaine *et al.* 2011[17]
	b	96.95<br>5.45		70.91<br>3.9	67.17<br>3.73	---	61.7<br>3.7/3.8	
	c	94.55<br>4.90	71.94<br>3.63	---	67.40<br>3.5	---	61.75<br>3.76	Archbald et al. 1981[18]
	d	94.85<br>5.17	---	---	---	---	---	Fontaine *et al.* 2011[17]
	e	89.23<br>6.05	---	74.85<br>4.12	71.3<br>4.40/3.6	---	62.20<br>3.80, 3.90	

	f	94.77<br>4.94	71.87<br>3.92	---	68.82<br>3.79	---	63.85<br>3.7	
	g	88.64<br>5.87	---	74.35<br>3.5	71.21<br>3.64	---	62.25<br>3.85	
Glucose (Glc)	a (α)	92.94<br>5.20	72.40<br>3.50	72.40<br>3.50	70.60<br>3.40	---	61.56<br>3.70, 3.80	Archbald *et al.* 1981[18]
	b (β)	b (β)	96.84<br>4.63	74.90<br>3.25	76.60<br>3.50	70.50<br>3.40	---	
GalNAc		96.87<br>4.61	55.61<br>3.61	74.9<br>3.25	76.6<br>3.50	70.5<br>3.34	61.65<br>3.7, 3.88	
GalN		92.94<br>5.22	54.94<br>3.69	72.4<br>3.5	70.9<br>3.4	---	61.4<br>3.7/3.83	
Galf	a	108.51<br>5.05	81.8<br>4.13	77.65<br>4.07	83.58<br>4.07	71.68<br>3.84	63.75<br>3.67/3.84	
	b	107.67<br>5.21	82.13<br>4.13	77.27<br>4.07	83.01<br>4.0	71.13<br>3.9	63.75<br>3.67/3.84	

Table S10. ^{1}H and ^{13}C chemical shifts of rigid polysaccharides from ^{1}H-detection experiments. For each carbon site, the ^{13}C and ^{1}H chemical shifts are shown in the top and bottom rows, respectively. The referencing scale is TMS scale for ^{13}C, and DSS for ^{1}H.

Carbohydrates	C1	C2	C3	C4	C5	C6	Reference
β -1,3-glucan	103.6<br>5.35	74.4<br>3.9	86.8<br>3.9	68.4<br>3.85	77.6<br>3.7	61.4<br>4.2	Shim *et al*. 2007
α-1,3-glucan type-a	101.1<br>5.7	71.9<br>3.5,3.8,<br>4.1,4.5	84.9<br>3.9	69.9<br>4.0	71.6<br>3.5, 3.8, 4.1, 4.5	60.9<br>4.15	Bhanja et al.2014<br>Yarava *et al*. 2025
α-1,3-glucan type-d	101.1<br>5.7	---	84.41<br>4.40	---	---	---	Yarava *et al*. 2025
α-1,3-glucan type-e	100.76<br>5.4	---	84.38<br>3.57	---	---	---	Yarava *et al*. 2025
α-1,3-glucan type-f	101.42<br>5.2	---	84.38<br>3.57	---	---	---	
α-1,3-glucan type-g	101.03<br>6.58	---	84.37<br>4.81	---	---	---	
α-1,3-glucan type-h	101.16.0		84.9<br>3.9				
Chitosan	102.2<br>4.7, 4.3,<br>5.87	55.9<br>4.21	73.0<br>4.15	80.4<br>5.0	75.7<br>3.85	-	
Chitin type-a	103.9<br>5.35	55.3<br>4.5	73.7<br>4.15	83.5<br>3.85	75.3<br>3.9	61.7<br>4.2	Fernando *et al.* 2021

Table S11. Experimental parameters used for both WT and mutant A. fumigatus. Rigid region of the cell wall was characterized using CP based experiments on 600 MHz (14.1T) spectrometer using 1.3mm triple resonance MAS probe with the MAS frequency of 60 kHz.

Expt.	CP (μs)		NS	d1 (s)	td2	td1	td3	aq2 (ms)	aq1 (ms)	aq3 (ms)	DIPSI-3 (ms)	RFDR mixing (ms)	Expt. Time (h)
	t_{cp1}	t_{cp2}											
2D hCH	500 (HC-CP)	50 (CH-CP)	32	2	1764 (^{1}H)	448 (^{13}C)	-	14.9	7.46	-	-	-	8h 34m
2D hChH (RFDR)	500 (HC-CP)	500 (CH-CP)	32	2	1764 (^{1}H)	448 (^{13}C)	-	14.9	7.46	-	-	0.533	8h 28m

Table S12. **Experimental parameters used for both *A. fumigatus* WT and mutant samples.** Mobile region of the cell wall was characterized using scalar-coupling (*J*) based experiments on 800 MHz (18.8T) spectrometer using 3.2 mm triple resonance MAS probe with the MAS frequency of 15 kHz.

Experiment	MAS	d1	NS	td2	td1	td3	aq2 (ms)	aq1 (ms)	aq3 (ms)	Decoupling/ Water suppression	*J*-evolution (ms)	DIPSI-3 (ms)	Expt. Time (h)
										SPINAL-64 (rf 71.429 kHz)			
2D hCCH TOCSY (DIPSI-3)	15	2	32	2614 (^{1}H)	512 (^{13}C)	1	39.9	10.2	-	WALTZ-16 (rf 17 kHz) MISSISSIPI (total duration) 40 ms (rf 25.994 kHz	1.78 (τ_1) 1.19 (τ_2	25.5	9h 42m
3D hCCH TOCSY (DIPSI-3)	15	2	8	2614 (^{1}H)	128 (^{13}C)	128 (^{13}C)	39.9	2.56	2.56				77h 53m

Supplementary References

1 Malavazi, I. & Goldman, G. H. in *Host-Fungus Interactions: Methods and Protocols* 99-118 (Springer, 2012).

2 Shim, J. H. *et al.* Antitumor Effect of Soluble b-1, 3-Glucan from Agrobacterium sp. R259 KCTC 1019. *J. Microbiol. Biotechnol.* **17**, 1513-1520 (2007).

3 Bhanja, S. K. *et al.* Water-insoluble glucans from the edible fungus Ramaria botrytis. *Bioactive carbohydrates and dietary Fibre* **3**, 52-58 (2014).

4 Fernando, L. D. *et al.* Structural polymorphism of chitin and chitosan in fungal cell walls from solid-state NMR and principal component analysis. *Frontiers in Molecular Biosciences* **8**, 727053 (2021).

5 Lowman, D. W. *et al.* New insights into the structure of (1→ 3, 1→ 6)-β-D-glucan side chains in the Candida glabrata cell wall. *PloS one* **6**, e27614 (2011).

6 Shim, J.-H. *et al.* Antitumor Effect of Soluble ${\beta} $-1, 3-Glucan from Agrobacterium sp. R259 KCTC 1019. *Journal of microbiology and biotechnology* **17**, 1513-1520 (2007).

7 Fairweather, J. K., Him, J. L. K., Heux, L., Driguez, H. & Bulone, V. Structural characterization by ^{13}C-NMR spectroscopy of products synthesized in vitro by polysaccharide synthases using ^{13}C-enriched glycosyl donors: application to a UDP-glucose:(1→ 3)-b-d-glucan synthase from blackberry (Rubus fruticosus). *Glycobiology* **14**, 775-781 (2004).

8 Saitô, H., Ohki, T. & Sasaki, T. A 13C-nuclear magnetic resonance study of polysaccharide gels. Molecular architecture in the gels consisting of fungal, branched (1→ 3)-b-D-glucans (lentinan and schizophyllan) as manifested by conformational changes induced by sodium hydroxide. *Carbohydr. Res.* **74**, 227-240 (1979).

9 Chakraborty, A. *et al.* A molecular vision of fungal cell wall organization by functional genomics and solid-state NMR. *Nature communications* **12**, 6346 (2021).

10 Latge, J.-P. *et al.* Chemical and immunological characterization of the extracellular galactomannan of Aspergillus fumigatus. *Infection and immunity* **62**, 5424-5433 (1994).

11 Fontaine, T. *et al.* Galactosaminogalactan, a new immunosuppressive polysaccharide of Aspergillus fumigatus. *PLoS pathogens* **7**, e1002372 (2011).

12 Archbald, P. J., Fenn, M. D. & Roy, A. B. 13C-NMR studies of D-glucose and D-galactose monosulphates. *Carbohydrate Research* **93**, 177-190 (1981).

13 Latge, J. P. *et al.* Chemical and immunological characterization of the extracellular galactomannan of Aspergillus fumigatus. *Infect. Immun.* **62**, 5424-5433 (1994).

14 Chakraborty, A. *et al.* A molecular vision of fungal cell wall organization by functional genomics and solid-state NMR. *Nat. Commun.* **12**, 6346 (2021).

15 Kuraoka, T., Yamada, T., Takatsutsumi, Y., Ogawa, Y. & Kobayashi, H. Anomeric Proton and Carbon (H1-C1) NMR Chemical Shifts of Antigenic Mannans Obtained from Pathogenic Yeast Candida tropicalis. *Adv. Microbiol.* **11**, 296-301 (2021).

16 Kuraoka, T., Ishiyama, A., Oyamada, H., Ogawa, Y. & Kobayashi, H. Presence of O-glycosidically linked oligosaccharides in the cell wall mannan of Candida krusei purified with Benanomicin A. *FEBS Open Bio.* **9**, 129-136 (2018).

17 Fontaine, T. *et al.* Galactosaminogalactan, a New Immunosuppressive Polysaccharide of Aspergillus fumigatus. *PLoS Pathog.* **7**, e1002372 (2011).

18 Archbald, P. J., Fenn, M. D. & Roy, A. B. 13C-N.M.R. studies of D-glucose and D-galactose monosulphates. *Carbohydr. Res.* **93**, 177-190 (1981).
